# Evaluation of Cytotoxic Potential of Newly Synthesized Antiviral Aminopyrazoloquinoline Derivatives

**Published:** 2007-09

**Authors:** Jamal M. Arif, Mohammed Kunhi, Manogaran P. Subramanian, Adnan A. Bekhit, Ola A. El-Sayed, Khalid Al-Hussein, Hassan Y. Aboul-Enein, Fahad M. Al-Khodairy

**Affiliations:** 1 *Department of Biological and Medical Research, King Faisal Specialist Hospital and Research Center, P.O. Box 3354, Riyadh 11211, Saudi Arabia;*; 2 *Pharmaceutical and Medicinal Chemistry Department, National Research Centre, Dokki, Cairo 12311, Egypt*

**Keywords:** aminopyrazoloquinoline, apoptosis, DNA damage, MCF-7, MDA-MB-231, MCF-12, MCF-10A

## Abstract

In the present study, we screened newly synthesized antiviral aminopyrazoloquinoline derivatives for cytotoxic potential in human normal and breast cancer cell lines using apoptosis as biomarker. These derivatives and the well known antiviral drug, acyclovir, were incubated with the normal (MCF-10A, MCF-12A) and cancer (MCF-7, MDA-MB-231) cell lines at 10, 50 and 100 μM for 72 h at 37°C. Both the parent compounds and their sugar derivatives were found to be differentially cytotoxic in various cell lines. MCF-7 cells were more or less completely resistant to all these compounds while MDA-MB-231 cells were significantly killed by apoptosis. The methoxy derivative of aminopyrazoloquinoline (compound 3) was found to be the most cytotoxic in the normal breast epithelial cell lines (MCF-10A and MCF-12A) and MDA-MB-231 cell lines at 100 μM killing over 90% of the cells with up to 80% apoptosis. Interestingly MCF-7 cells showed only up to 50% killing at 100 μM dose with less than 20% apoptosis. Acyclovir did not cause any cytotoxicity, apoptosis or cell cycle arrest in any of the cells lines at the doses tested. Our results suggest that the newly synthesized antiviral compounds have an associated risk of being cytotoxic compared to the acyclovir.

## INTRODUCTION

**Figure 1:**
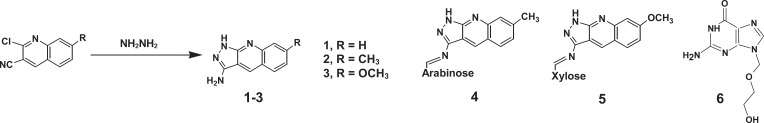
Chemical structures of aminopyrazoloquinoline derivatives. **Compound 1:** 3-amino-1H-pyrazolo[3,4-b]quinoline; **Compound 2:** 3-amino-7-methyl-1H-pyrazolo[3,4-b]quinoline; **Compound 3:** 3-amino-7-methoxy-1H-pyrazolo[3,4-b]quinoline; **Compound 4:** aldehydo-D-arabinose{7-methyl-1H-pyrazolo[3,4-b]quinoline-3-yl}imine; **Compound 5:** aldehydo-D-xylose{7-methoxy-1H-pyrazolo[3,4-b]quinoline-3-yl}imine; **Compound 6:** acyclovir.

Acyclovir had been the drug of choice for several decades; however, the emergence of acyclovir-resistant Herpes Simplex Virus (HSV) isolate in the immunocompromised patients and organ or bone marrow recipients has led to the discovery of new antiviral compounds (3-5, 9, 10). Keeping this in mind, we synthesized several pharmacologically active aminopyrazoloquinoline derivatives with and without sugar moieties, which have been shown to possess different degree of antiviral activities against HSV-type 1 (HSV-1) (1, 2). The 3-amino-7-methoxy-1H-pyrazolo [3, 4-b] quinoline (compound 3, Fig. 1) was found to be equally effective antiviral compound as acyclovir at equimolar basis (2). Although aldehydo-sugar derivatives of pyrazoloquinoline (compounds 4 and 5, Fig. 1) inhibited the HSV-1 replication, their precursors were more effective anti-HSV-1 (2).

In order to provide the new generation of antiviral drugs with the least possible cytotoxicity, we evaluated their cytotoxic potential using apoptosis in various human normal and breast carcinoma cell lines. Apoptosis is a well-established and recognized biomarker to screen the new compounds for cytotoxicity (6, 11-13). On the basis of our published report (2) regarding the antiviral activities of these aminopyrazoloquinoline precursors and their aldehydo-sugar derivatives, we selected only the most effective antiviral derivatives for evaluation of cytotoxic potential.

## INVESTIGATIONS, RESULTS AND DISCUSSION

**Figure 2:**
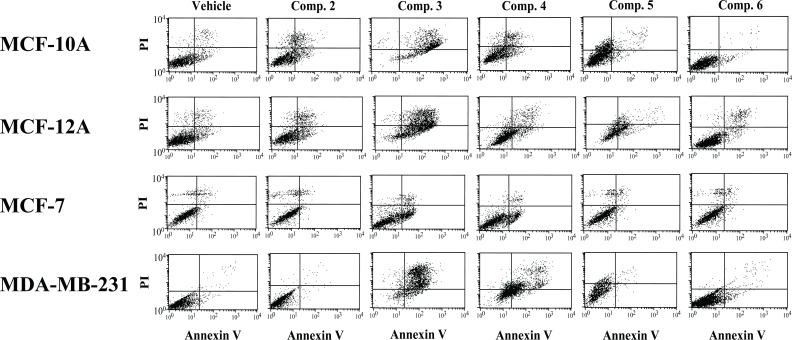
Flow cytometric analysis showing the distribution of various cells treated with only 100 µM aminopyrazoloquinoline (compounds 1-5) and acyclovir (compound 6). The cells were analyzed by flow cytometry using CellQuest Pro software.

**Figure 3:**
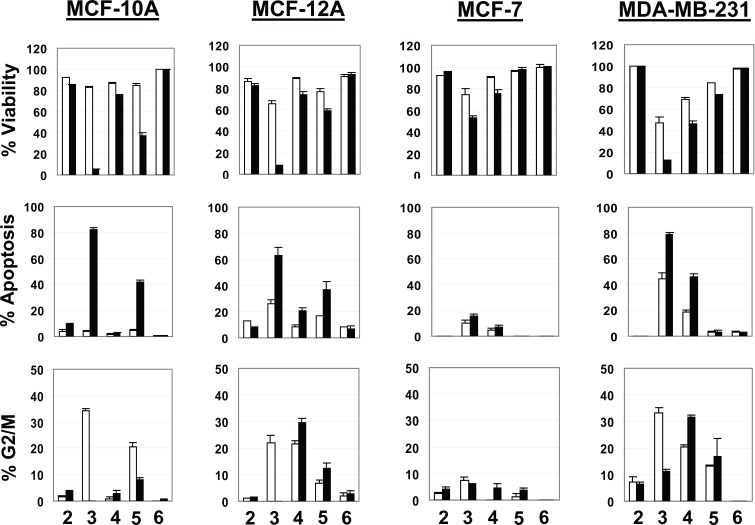
Percentage of viable, apoptotic and G2/M arrested cells following treatment with the test compounds at various concentrations. The data was normalized against the control (100% for viability or 0% for apoptosis). The empty bars represent 50 μM while solid bars show 100 μM concentrations of the compounds. The values are represented as mean ± SD (n=3).

Since the inception of apoptosis in 1842 and its revival almost 30 years ago, numerous publications using apoptosis have appeared in the literature (6). Apoptosis is a sophisticated and complex pathway involving more than 100 proteins and has been implicated in the screening of numerous drugs and compounds for cytotoxic and anticancer potentials using various cancer and normal cell lines (6, 11-13). In the present study, we evaluated aminopyrazoloquinoline derivatives for cytotoxic potential using apoptosis as a marker. At relatively lower concentration (10 μM), none of the compounds showed any appreciable cell death, apoptosis or cell cycle arrest in the normal (MCF-12A) or breast cancer (MCF-7, MDA-MB-231) cells (data not shown). However, increasing the concentration to 50 μM, both compound 1 and its methylated derivative (compound 2, Fig. 1) were slightly effective in inducing apoptosis in the normal and cancer cell lines. Due to unavailability of its sugar-derivative along with its mild anti-HSV-1 activity, it was not further tested. However, compound 2 at 50 μM also caused insignificant apoptosis in the normal MCF-10A and MCF-12A cell lines while no effect was observed in either of the cancer cells (Figure 3). Increasing the dose to 100 μM did not significantly change the scenario regarding the apoptosis and cell cycle arrest (Figures 2 and 3).

However, the methoxy derivative of aminopyrazoloquinoline (compound 3, Fig. 1) that showed significant anti-HSV-1 activity (2) was also maximally cytotoxic. At 50 μM, it caused 20-30% cell deaths in the normal cells while 30-55% cancer cells were killed (Figure 3). The cells arrested in G2/M phase were 20-35% for normal cells with ~30% apoptosis in MCF-12A; MCF-10A showed only ~5% apoptosis. However, in MDA-MB-231 cell lines, approximately 35% cells were arrested in G2/M phase with >45% apoptotic cells. Interestingly, at 50 μM concentration, MCF-7 cells were the least affected with only <10% apoptosis. Further, at 100 μM concentration, this compound killed >90% of the normal (MCF-10A, MCF-12A) and cancer (MDA-MB-231) cells with up to 80% apoptosis; however, only 50% cytotoxicity was observed in MCF-7 cells along with <20% apoptosis (Figures 2 and 3). Accordingly, both estrogen receptor alpha negative (ER-) cells (MCF-10A and MDA-MB-231) showed maximum 80% apoptosis. The estrogen receptor alpha positive (ER^+^) normal MCF-12A cells showed about 60% apoptosis; however, the ER^+^ malignant MCF-7 cells tolerated this compound very well. Other biomolecular factors such as deficiencies of caspase-3 in MCF-7, BCL_2_ in MCF-12A, p16 in MCF-10A and presence of mutant p53 in MDA-MB-231 may be playing significant roles in cell-specific differential responses. However, the resistance of caspase-3 deficient MCF-7 cell lines (8) to any of these compounds compared to other cell lines regardless of their non-malignant or malignant nature suggests involvement of caspase-3 in the induction of apoptosis by these derivatives.

Further, the apoptotic potential of these synthetic compounds could be manipulated by substitution of various functional groups. The arabinose derivative (compound 4, Fig. 1) showed similar responses in MCF-10A, MCF-12A and MCF-7 cells as observed with the parent compound 2 except significant numbers (20-30%) of MCF-12A cells were arrested in G2/M phase (Figures 2 and 3). However, addition of arabinose sugar significantly affected the MDA-MB-231 cells. The arabinose analog killed 40-60% of the cells at both 50 and 100 μM doses with 20-50% apoptosis. Interestingly 20-30% of MDA-MB-231 cells were also arrested in G2/M phase. In contrast, the cytotoxic action of the most toxic methoxy derivative (compound 3, Fig. 1) of aminopyrazoloquinoline was drastically inhibited by the addition of xylose sugar (compound 5, Fig. 1). Surprisingly the apoptotic potential of compounds 3 and 5 coincided with their respective anti-HSV-1 activity (2) where addition of xylose sugar drastically reduced anti-HSV-1 activity of compound 3. At 50 μM dose, both normal and cancer cells showed no signs of cytotoxicity by compound 5 (Fig. 1), however, increasing the dose to 100 μM killed about 50% of the normal cells (MCF-10A and MCF-12A) along with 40% apoptosis. Surprisingly, both MCF-7 and MDA-MB-231 were almost completely resistant to the cytotoxic action of compound 5 at even 100 μM dose. Most interestingly, the widely used antiviral drug acyclovir did not cause any significant apoptosis, cytotoxicity or cell arrest in either of the cell lines at any concentration. Further, role of structure modifications in differential responses by aminopyrazoloquinoline analogs cannot be ruled out.

In conclusion, our results suggest that the most effective anti-HSV-1 compound 3 was also found to be the most cytotoxic to the breast cancer or normal epithelial cell lines at ≥50 μM doses. In contrast, the widely used anti-HSV-1 drug, acyclovir, did not cause cytotoxicity, apoptosis or cell cycle arrest in any of these cell lines at the doses tested. Although the new antiviral aminopyrazoloquinoline derivatives have great and comparable potential to acyclovir for treatment of HSV-1 infection especially in the resistant immunocompromised patients, bone marrow and organ transplant recipients, these new antiviral compounds have an associated risk of cytotoxicity as a side effect.

## EXPERIMENTAL

### Chemicals and cell lines

The aminopyrazoloquinoline derivatives were synthesized as described earlier (1, 2). The chemical structures for these compounds are shown in Figure 1. Human breast carcinoma cell lines (MCF-7, MDA-MB-231) and normal human breast epithelial cell lines (MCF-10A, MCF-12A) were purchased from American Type Culture Collection (Rockville, MD, USA). All the cell culture reagents and media were obtained from Sigma Chemical Company (St. Louis, MO, USA) except cholera toxin, which was purchased from Miller and Miller (Chemical) Ltd. (London, UK). Vybrant apoptosis assay kit 2 was purchased from Molecular Probes Inc. (OR, USA).

### Cell culture and treatments

MCF-7 and MDA-MB-231 cells were cultured in RPMI1640 media using 10% calf serum to its confluence. The MCF-10A and MCF-12A were cultured in Dulbecco’s modified Eagle’s medium/Ham’s nutrient mixture F12 (1:1, v/v) supplemented with cholera toxin (100 μg/ml), epidermal growth factor (20 μg/ml), insulin (10 μg/ml), hydrocortisone (0.5 μg/ml) and 5% calf serum. For apoptosis and cell viability assays, the cells were treated with the test compounds (10, 50 and/or 100 μM) in triplicate and incubated in the CO_2_ incubators with 5% CO_2_ for 72 h.

### Cell viability and induction of apoptosis

Treated cells were trypsinized, harvested and washed in cold phosphate-buffered saline and centrifuged followed by staining with annexin V and propidium iodide in annexin-binding buffer. After 15 min incubation at room temperature, the fluorescence was measured using the flow cytometer (FACScan, Becton Dickenson, CA, USA). The results were analyzed using Cell Quest Pro software and represented as percentage of normal and apoptotic cells at various stages. Simultaneously, treated cells were also stained for nuclear DNA and sub-G_1_ apoptotic population was analyzed (7).

### Statistical analysis

The values represent mean ± SD (n=3). The statistical significance was determined by the student *t* test and the value *p*<0.05 was considered significant.

## References

[1] Bekhit AA, El-Sayed OA, Aboul-Enein HY, Siddiqui YM (2004). Synthesis of aldehydo-sugar derivatives of pyrazoloquinoline as inhibitors of herpes simplex virus type 1 replication. J. Enzyme. Inhib. Med. Chem.

[2] Bekhit AA, El-Sayed OA, Aboul-Enein HY, Siddiqui YM (2005). Evaluation of some pyrazoloquinolines as inhibitors of herpes simplex virus type 1 replication. Arch. Pharm. (Weinheim).

[3] Danve-Szatanek C, Aymard M, Thouvenot D, Morfin F (2004). Surveillance network for herpes simplex virus resistance to antiviral drugs: 3-year follow-up. J. Clin. Microbial.

[4] Darville JM, Ley BE, Roome AP, Foot AB (1998). Acyclovir-resistant herpes simplex virus infections in a bone marrow transplant population. Bone Marrow Transplant.

[5] Englund JA, Zimmerman ME, Swierkosz EM, Goodman JL (1990). Herpes simplex virus resistant to acyclovir. Ann. Intern. Med.

[6] Gosslau A, Chen KY (2004). Nutraceticals, apoptosis, and disease prevention. Nutrition.

[7] Krishan A (1975). Rapid flow cytofluormetric analysis of mammalian cell cycle by propidium iodide staining. J. Cell Biol.

[8] Liang Y, Yan C, Schor NF (2001). Apoptosis in the absence of caspase 3. Oncogene.

[9] Ljungman P, Ellis MN, Hackman RC, Shepp DH (1990). Acyclovir-resistant herpes simplex virus causing pneumonia after marrow transplantation. J. Infect Dis.

[10] Morfin F, Thouvenot D (2003). Herpes simplex virus resistance to antiviral drugs. J. Clin. Virol.

[11] Narayan S, Jaiswal AS, Kang D, Srivastava P (2004). Cigarette smoke condensate-induced transformation of normal human breast epithelial cells *in vitro*. Oncogene.

[12] Tseng E, Scott-Ramsay EA, Morris ME (2004). Dietary organic isothiocyanates are cytotoxic in human breast cancer MCF-7 and mammary epithelial MCF-12A cell lines. Exp. Biol. Med.

[13] Upadhyay S, Neburi M, Chinni SR, Alhasan S (2001). Differential sensitivity of normal and malignant breast epithelial cells to genistein is partly mediated by p21 (WAF1). Clin. Cancer Res.

